# Editorial Notes

**Published:** 1948

**Authors:** 


### Editorial Notes

**Vale atque ave.**

Our readers will join with the Committee of the Bristol Medico-Chirurgical Society in sincere acknowledgment of the untiring labours on their behalf, during thirty-nine years, of the retiring Editor, Professor J. A. Nixon. Editorial Secretary in 1909, he was appointed Assistant Editor in 1912 and Editor in 1926. A year ago the *Journal* offered him congratulations on the completion of twenty-one years in the chair. It is easy to forget what a mass of work this means, and how great the demands on the time and energies of a busy man. The regular monthly meetings of the Editorial Committee are a small part only of this ; but the first at which he presided, in March 1926, was No. 348 and the last No. 516! And in addition he contributed a full share of articles, especially notable being those on subjects connected with Medical History.

Thanks are due also to Professor A. Rendle Short, who has served on the Journal Committee since 1933. We trust that both these distinguished colleagues will continue their help and support.

We wish to congratulate our readers on securing the services of Dr. N. S. Craig as Assistant Editor, an appointment involving more work than glory, and hope that we shall long continue to gather the fruits of his labours.

**The Quads.**

Bristol has recently been real news, in the journalist's sense. The generation now growing up, we suppose, when it hears the name will think at once, not of Cabot, *Treasure Island*, glass, milk or even the Brabazon—but of the quadruplets. We hope to publish an account of this event shortly: but understand that the film rights are no longer available.

**Subscribers.**

We reprint below a letter that has been sent to ex-students of Bristol University and to a number of practitioners in the Region. We ask readers to bring the *Journal* to the notice of colleagues and remind those who have to resign from the Society that they can still keep in touch with local affairs by subscribing to the *Journal.* Names and addresses of prospective subscribers should be sent to the Treasurer, Dr. Frederick Sutton, F.R.C.P., 1 Pembroke Road, Bristol 8.

"Although *The Bristol Medico-Chirurgical Journal* is one of the very few provincial journals that continued to appear, the recent war was not without effects on our connection. We feel that the time has now come to renew old associations and to replace the losses of time and circumstance. Not many years ago the *Journal* circulated widely throughout the country, particularly in the South-West, and regularly recorded the activities of many Medical Societies in this area. With the inauguration of the new Regional organization we trust that we shall once again be the *Journal*, not merely of Bristol, but of the whole South-Western Region.

"All this, however, requires help from outside Bristol. There are many lectures, addresses and discussions of great clinical or local interest that ought to be recorded: there are many medical events of special local importance. In order to publish these we need the help of colleagues; and particularly invite anyone who is willing to become "Local Correspondent," or whose fellows can persuade him to undertake this rather thankless task, to keep us informed of what is being said or done.

"Will you bring the *Journal* to the notice of colleagues, particularly of members of local Medical Societies? A limited number of copies of the first issue for this year can be supplied for this purpose on request, or will be sent direct to any colleagues who you think will be interested. We shall value especially any comments or suggestions to enhance the value of our publication and all regular or occasional information: original articles are welcomed (we regret that for the present the limit is about 2,000 words). And we wish to add that, for the former Bristol student, the *Journal* affords an excellent means of keeping in touch with his old school and with local current events."

